# Coupling Miniaturized Stir Bar Sorptive Dispersive Microextraction to Needle-Based Electrospray Ionization Emitters for Mass Spectrometry: Determination of Tetrahydrocannabinol in Human Saliva as a Proof of Concept

**DOI:** 10.1021/acs.analchem.4c01297

**Published:** 2024-05-14

**Authors:** Andreu L. López-Juan, Jaime Millán-Santiago, Juan L. Benedé, Alberto Chisvert, Rafael Lucena, Soledad Cárdenas

**Affiliations:** † GICAPC Research Group, Department of Analytical Chemistry, University of Valencia, Burjassot E-46100, Valencia, Spain; ‡ Affordable and Sustainable Sample Preparation (AS2P) Research Group, Analytical Chemistry Department, Instituto Químico para la Energía y el Medioambiente (IQUEMA), University of Córdoba, Campus of Rabanales, Marie Curie Building, Córdoba E-14071, Spain

## Abstract

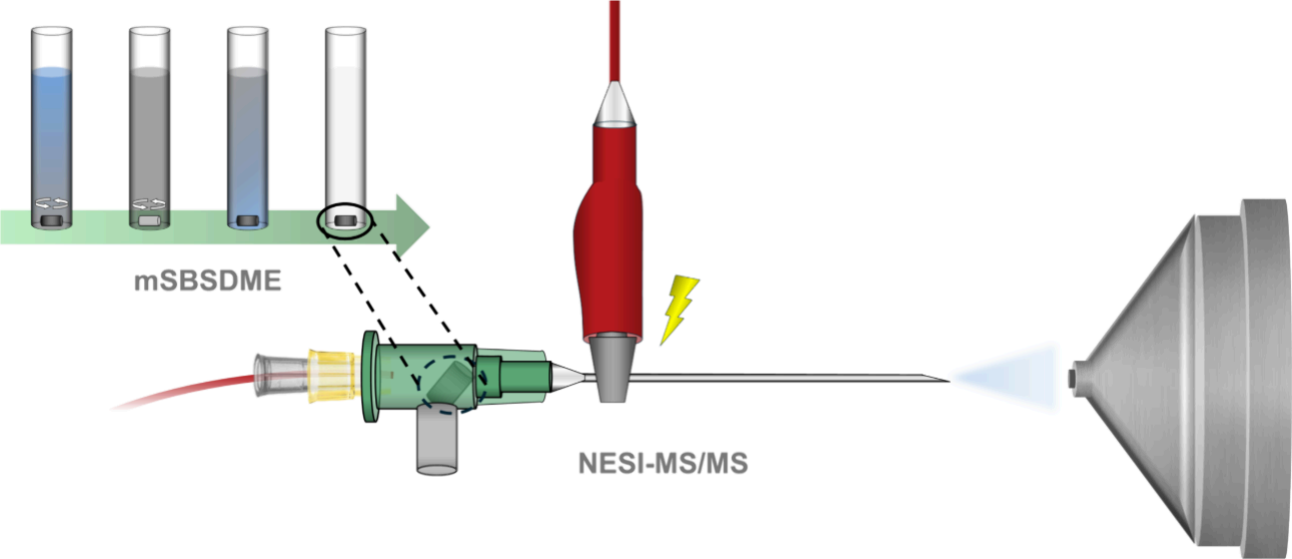

Direct coupling of sample preparation with mass spectrometry (MS) can speed up analysis, enabling faster decision-making. In such combinations, where the analysis time is mainly defined by the extraction procedure, magnetic dispersive solid-phase extraction emerges as a relevant technique because of its rapid workflow. The dispersion and retrieval of the magnetic sorbent are typically uncoupled stages, thus reducing the potential simplicity. Stir bar sorptive dispersive microextraction (SBSDME) is a novel technique that integrates both stages into a single device. Its miniaturization (mSBSDME) makes it more portable and compatible with low-availability samples. This article reports the direct combination of mSBSDME and MS using a needle-based electrospray ionization (NESI) emitter as the interface. This combination is applied to determine tetrahydrocannabinol in saliva samples, a relevant societal problem if the global consumption rates of cannabis are considered. The coupling requires only the transference of the magnet (containing the sorbent and the isolated analyte) from the mSBSDME to the hub of a hypodermic needle, where the online elution occurs. The application of 5 kV on the needle forms an electrospray on its tip, transferring the ionized analyte to the MS inlet. The excellent performance of mSBSDME-NESI-MS/MS relies on the sensitivity (limits of detection as low as 2.25 ng mL^–1^), the precision (relative standard deviation lower than 15%), and the accuracy (relative recoveries ranged from 87 to 127%) obtained. According to the results, the mSBSDME-NESI-MS/MS technique promises faster and more efficient chemical analysis in MS-based applications.

## Introduction

The direct combination of sample preparation strategies and mass spectrometry (MS) detection has emerged as a valuable alternative to classical analytical approaches that typically intercalate a separation technique (i.e., chromatography) between them.^1^ The bypass of the chromatographic separation can lead to faster analytical methods, increasing the number of samples that can be scrutinized. Different ambient ionization techniques have been proposed in these couplings, including direct analysis in real time,^2−4^ dielectric barrier desorption ionization,^5,6^ atmospheric solids analysis probe,^7^ extractive-liquid sampling electron ionization mass spectrometry,^8^ or substrate spray ionization (SSI).^9^ The latter technique is especially interesting due to its efficiency and simplicity. Also, it is based on a well-established ionization mode (electrospray imaging, ESI) in bioanalysis. In SSI, the sample is placed in a solid substrate and typically dried after loading. Subsequently, a solvent is added to the substrate. The application of a high voltage generates a spray in the substrate tip, thus transferring the ionized analytes to the MS inlet. SSI can be combined with microextraction techniques in two ways. In liquid-phase microextraction, the isolation of the analytes is performed offline, and the liquid extract is deposited over the substrate for the analysis.^10^ In solid-phase microextraction, the substrate integrates the isolation and analysis steps. Different solid substrates have been proposed for this integration, including paper,^11^ wooden tips,^12−14^ needles,^15,16^ tapes,^17,18^ or coated blades.^19,20^

As SSI measurements are typically fast, the analysis time in microextraction-SSI couplings mainly depends on the sample preparation step. Unlike static techniques, dispersive solid-phase extraction (DSPE)^21^ speeds up the isolation of the analytes by the efficient dispersion of the sorbent into the sample. The overall analysis time is further decreased when magnetic sorbents are used since they can be quickly and easily retrieved by applying an external magnetic field. Magnetic-based DSPE has been successfully coupled to SSI in several ways. In the simplest one, the DSPE is offline performed, and the eluate containing the analytes is deposited over a substrate for their analysis.^22^ The integration of the elution and analysis in a single step has also been proposed. Geballa-Koukoula et al.^23^ used a magnetic sorbent for an offline DSPE, and after extraction, it was suspended in water and pipetted onto the tip of a noncoated blade. Then, the sorbent was fixed with an external magnet, and a solvent was added to perform the elution and ESI directly in front of the MS inlet. Other authors have proposed the so-called internal extractive electrospray ionization mass spectrometry (iEESI) technique for such coupling.^24−26^ In iEESI, the sorbent is dispersed into the sample in an appropriate vessel, and the dispersion is taken with a syringe. The magnetic sorbent is manually retained in the syringe barrel with the aid of an external magnet, whereas the leftover sample is discarded. Then, the sorbent is washed with an appropriate solvent to remove potential interferences. Finally, the elution solvent is loaded into the syringe and gently shaken to desorb the target compounds, and the resulting dispersion is pumped through a capillary for ESI, where an external magnet is placed to prevent the arrival of the sorbent to the MS inlet. In these approaches, the dispersion and retrieval of the sorbent particles are performed in independent and manual steps.

Stir bar sorptive dispersive microextraction (SBSDME),^27^ unlike conventional DSPE, integrates the dispersion and retrieval of the magnetic sorbent in a single device. The technique uses a bar-shaped magnet, where the magnetic sorbent is deposited. At high stirring rates, the rotational force surpasses the magnetic attraction, dispersing the sorbent into the sample. When the stirring is over, the magnetic attraction prevails and the sorbent is captured by the bar. The technique has been recently scaled down in the so-called miniaturized stir bar sorptive dispersive microextraction (mSBSDME),^28^ which presents important advantages. It is adapted to process low-availability samples (e.g., saliva and the follicular fluid)^28,29^ because of the reduction of the extraction device. It decreases the sorbent and solvent requirements, thus reducing waste generation. Most notably, for bioanalysis and as a consequence of the reduction of the physical space, different samples can be treated simultaneously, thus providing a higher sample throughput and also better portability.

In this article, mSBSDME is combined for the first time to direct MS analysis using a needle-based electrospray ionization (NESI) emitter as the interface. After mSBSDME, the magnetic stir bar containing the sorbent is loaded into the hub of a hypodermic needle, which is online eluted, forming an electrospray in front of the MS inlet.

As a proof of concept to show the applicability of the presented mSBSDME-NESI-MS/MS approach, Δ^9^-tetrahydrocannabinol (THC), the main psychoactive substance present in cannabis, is determined in human saliva samples. A composite material made of cobalt ferrite magnetic nanoparticles (MNPs) entrapped into a poly(divinylbenzene-*co*-*N*-vinylpyrrolidone) copolymer (CoFe_2_O_4_@p(DVB-*co*-NVP)) is used as a sorbent to promote the interaction with THC through hydrophobic and π–π interactions and hydrogen bonding. It should be said that cannabis remains by far the most commonly consumed illicit drug in Europe. Around 8% of European adults (aged 15 to 64) are estimated to have used cannabis in 2022 (22.6 million), and around 1.3% are estimated to be daily or almost daily users.^30^ The widespread abuse of cannabis may have long-term consequences, such as the development of some respiratory and cardiovascular disorders and specific psychiatric disorders.^31^ In addition, the legalization of recreational cannabis in some nations increased the need of monitoring THC levels to promote public safety, prevent accidents, and ensure responsible cannabis use. In this context, rapid but reliable analysis is crucial to face these societal challenges. In this sense, saliva sampling is an accessible, noninvasive, and on-site screening biofluid for illicit drug consumption. Moreover, it is advantageous over urine and blood, as it is collected under direct observation, deterring adulteration and without requiring specialized collection by medical personnel.^32^

## Experimental Section

### Standards and Samples

Methanolic solutions of (−)-*trans*-Δ^9^-tetrahydrocannabinol (THC) 1 mg mL^–1^ (certified reference material), used as the standard, and (−)-*trans*-Δ^9^-tetrahydrocannabinol-*d*_3_ (THC-*d*_3_) 100 μg mL^–1^, used as the surrogate, were purchased from Sigma-Aldrich (St. Louis, MO, USA). The 1 mg mL^–1^ THC solution was properly diluted to obtain a 200 μg mL^–1^ solution in methanol and kept at −20 °C and protected from light. From this solution, a solution of 10 μg mL^–1^ in the same solvent was prepared monthly and kept at −20 °C and was covered from light. Similarly, 1 μg mL^–1^ THC-*d*_3_ was prepared monthly from the commercial solution and stored at −20 °C covered from light. Subsequently, from these intermediate solutions, working standard solutions of THC (7.5–450 ng mL^–1^) were freshly prepared in synthetic saliva (see the Supporting Information). Then, as described later for sample preparation, 250 μL of each of these working solutions was mixed, respectively, with 125 μL of acetonitrile (33% v/v), and then, 13.1 mg of NaCl (3.5% w/v) was added. Chemical structures and other relevant information are given in Table S1.

Saliva samples were collected in 15 mL glass centrifuge tubes from volunteers who did not eat or drink for at least 30 min before. The passive drooling method was used since swab collection methods (e.g., Salivette, from Sarstedt, Germany) could introduce variability and negatively affect results due to the hydrophobicity of the target compound.^33,34^ Due to the low stability of THC, samples were immediately analyzed or stored at −20 °C, and then, they were thawed and vortexed for homogenization just before analysis. For the removal of proteins, which cause ionic suppression, in triplicate, 250 μL of saliva was mixed with 125 μL of acetonitrile, and then, 13.1 mg of NaCl (3.5% w/v) was added. The solutions were stirred by vortexing for 10 s and centrifuged at 6000 rpm for 5 min, and the supernatants were then analyzed. This content of acetonitrile reduces the surface tension of water and facilitates the subsequent introduction of the sample solution into the flat-base glass inserts to perform mSBSDME. All volunteers gave written informed consent to participate in this study, which was approved by the Ethics Committee of the University of Valencia.

Methanol LC-MS-grade and formic acid reagent-grade from Merck (Darmstadt, Germany) were employed as eluent and ionization agents for NESI. Extrapure helium (>99.999%) provided by Air Liquide (Madrid, Spain) was used as a collision gas by collision-induced dissociation (CID).

Those reagents used for the synthesis of the sorbent material and those used for the optimization of the mSBSDME variables are described in the Supporting Information.

### Apparatuses

A ZX3 vortex mixer from VELP Scientifica (Usmate Velate, Italy) and an EBA 21 centrifuge from Hettich (Tuttlingem, Germany) were used during the protein precipitation step.

A lab-made multiextraction assembly consisting of a magnetic stirrer MX-3K (18 W, 0 to 3000 rpm) from Anzeser (Frankfurt am Main, Germany) with a 3D-printed support for up to 15,400 μL flat-base glass inserts (31 mm height × 4 mm i.d.) from Labbox (Barcelona, Spain) was used to carry out the mSBSDME.^28^ Cylindrical NdFeB magnets (3 mm length × 2 mm diameter, 45 MGO) from Supermagnete (Gottmadingen, Germany) were used to disperse and collect the magnetic sorbent from the donor solution.

A hypodermic needle (0.8 mm height × 40 mm length, 21 gauge) from Becton Dickinson and Company (Huesca, Spain) was used as an ESI emitter device.

A linear trap quadrupole (LTQ) Orbitrap XL hybrid mass spectrometer from Thermo Fisher Scientific (Waltham, MA, USA) working with an ion trap analyzer was used.

Those apparatuses used for the synthesis of the sorbent material and those used for the optimization of the mSBSDME variables are described in the Supporting Information.

### Miniaturized Stir Bar Sorptive Dispersive Microextraction Coupled to Needle-Based Electrospray Ionization Emitters

The mSBSDME procedure was performed by weighing 0.5 mg of the magnetic composite into a 400 μL flat-base glass insert containing a neodymium stir bar. Then, 350 μL of the previously prepared standard, or sample, solutions and 3 μL of the 1 μg mL^–1^ surrogate standard solution were introduced. High magnetic stirring was applied for 3 min. Once the extraction was completed, the stirring was halted, and the magnetic sorbent containing the target analytes was magnetically collected on the stir bar. Subsequently, the donor phase was carefully discarded with the aid of a gel loading pipet tip, and 350 μL of a 1% v/v acetonitrile solution in water was added into the extraction device and discarded in the same way to wash the sorbent.

To carry out the NESI-MS/MS, first, the sorbent-coated stir bar was carefully transferred into the hub of the hypodermic needle by decanting the extraction vial and with the help of an external magnet (5 mm length × 5 mm diameter). The position of the stir bar was fixed with the external magnet to prevent it from being attracted to the needle, thus blocking the flow of the eluent/ionization agent. An alligator clip clamped to the metallic hypodermic needle close to the hub was used to later apply the needed voltage to form the electrospray. It also conferred firmness to the proposed interface.

Subsequently, a 500 μL Hamilton syringe was filled with methanol containing 0.1% v/v formic acid, and then, it was placed in a syringe pump and connected to the needle hub by using a polyether ether ketone (PEEK) tube and a 10 μL cut pipet tip fitted into another 100 μL cut pipet tip. The eluent/ionization agent was automatically pumped at a flow rate of 30 μL min^–1^ to elute the target compounds.

**Figure 1:**
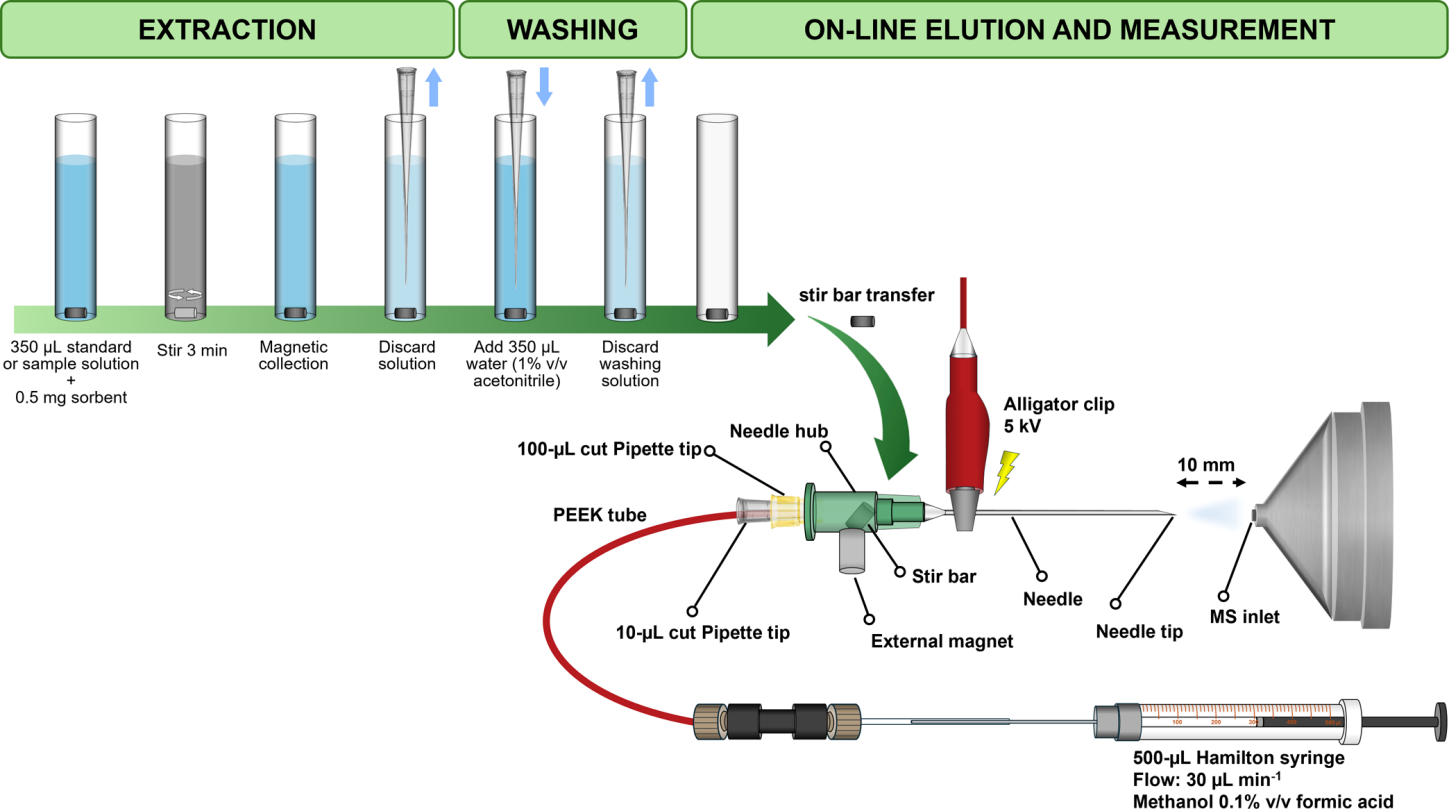
Schematic diagram of the proposed mSBSDME-NESI-MS/MS procedure.

Figure 1 shows a schematic diagram of the proposed mSBSDME-NESI-MS/MS procedure displaying the NESI assembly in detail.

The run time was 5 min. The LTQ MS detector was operated in the positive mode by multiple reaction monitoring (MRM). The voltage spray was set at 5 kV; the tube lens voltage was 130 V, and the capillary voltage and temperature were 35 V and 275 °C, respectively. The distance between the needle tip and the MS inlet was maintained at 10 mm, according to previous results.^35^ The *m*/*z* precursor → product ion transitions employed for quantification using 30 V as the collision energy were 315.3 → 193.1 and 318.3 → 196.1 for THC and THC-*d*_3_, respectively. The MS/MS isolation width was 1 amu in each transition.

## Results and Discussion

### Considerations on THC Retention on Polypropylene

**Figure 2:**
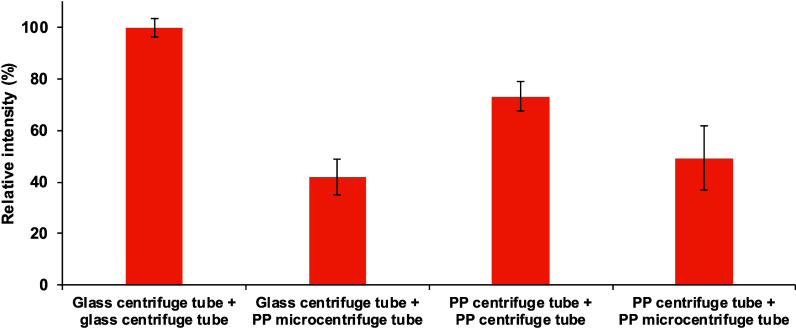
Effect of glass or PP tubes on the preparation and/or the storage for 1 h of a 10 ng mL^–1^ standard solution on the THC signal.

As known from previous works,^36,37^ THC can partially be adsorbed on polypropylene (PP) materials (such as micropipette tips, syringes, microcentrifuge tubes, etc.) when prepared in aqueous solutions due to its high hydrophobicity. As shown in Figure 2, when an aqueous standard solution of THC is prepared and/or stored for 1 h in PP centrifuge or microcentrifuge tubes, the signal decreases when compared to that using just glass centrifuge tubes. For this reason, glass material was used as much as possible throughout the whole work (e.g., Hamilton syringe and glass centrifuge tubes). Regardless, the presence of THC-*d*_3_ in both standard and sample solutions allows the use of plastic micropipet tips, which are handled more easily and faster than Hamilton syringes to take accurate volumes, since the unavoidable and undesirable interactions are the same for both the analyte and surrogate, and hence, they are corrected.

### Screening of the Extraction Variables

For the evaluation and selection of the critical variables involved in the extraction step, a screening study was performed. A Plackett–Burman design of 12 runs was applied to simultaneously screen the variables that significantly affect the analytical response.^38,39^ For this study, the selected extraction variables (and their ranges) were the sorbent amount (0.5–2.5 mg), the extraction time (1–10 min), the pH of the donor phase (2–10, 5 mM phosphate buffer), and the ionic strength (0–10% w/v NaCl, regardless of the ca. 0.25% w/v salt content provided by synthetic saliva). The Plackett–Burman design is shown in Table S3 (see the Supporting Information), and the statistical analysis was performed by employing the Minitab 18 (Minitab LLC, PA, USA) program as data treatment software and the chromatographic peak area of the analyte as the analytical response. All of the experiments were performed using 350 μL of a standard solution prepared in synthetic saliva containing 50 ng mL^–1^ of the target analyte. Liquid desorption of the analyte was carried out with 30 μL of methanol magnetically stirred for 30 s, and the extracts were analyzed by LC-MS/MS as described in the Supporting Information.

The results obtained were evaluated by analysis of variance (ANOVA) tests and represented by the Pareto chart of standardized effects (see Figure S1). As can be seen, the extraction time and the ionic strength presented a significant influence on the mSBSDME procedure, whereas the rest of the studied variables (i.e., the sorbent amount and pH) had negligible effects. Hence, only the formers needed to be optimized, selecting the minimum or nonadjusted values for the nonsignificant variables (i.e., a 0.5 mg sorbent amount and nonadjustment of pH).

### Optimization of the Extraction Variables

The extraction time and ionic strength were optimized to achieve the highest signals. To this regard, a response surface methodology (RSM) based on a two-factor Doehlert design was performed to establish the optimal values of the variables involved in the extraction procedure. The extraction time (1–10 min) and ionic strength (0–10% w/v NaCl) were studied by extracting 50 ng mL^–1^ of the target analytes in 350 μL of a standard solution prepared using synthetic saliva. As in the Plackett–Burman study, the extracts were analyzed by LC-MS/MS. The description of the statistics of the Doehlert design is included in the Supporting Information.

**Figure 3:**
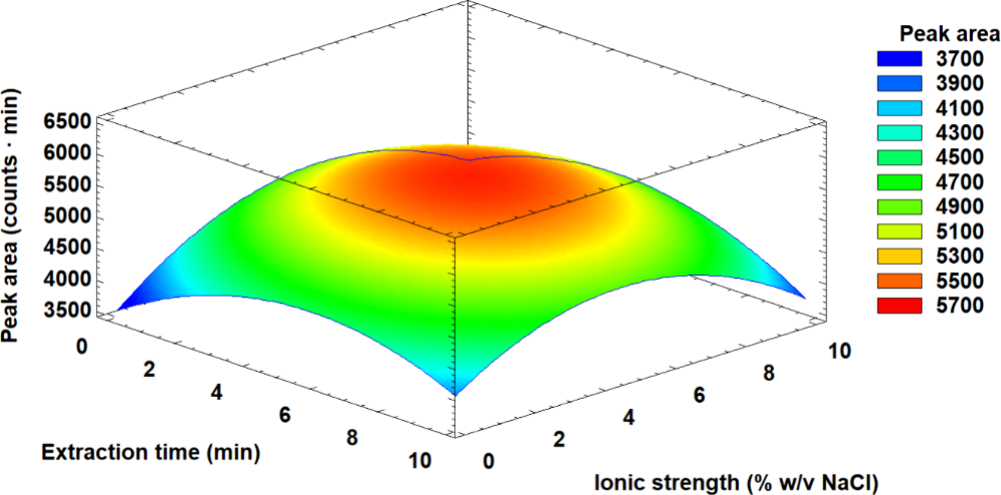
Response surface of the peak area function representing the relation between the studied critical variables: extraction time vs ionic strength.

As can be seen in Figure 3, the best results were obtained around a 6 min extraction time. However, 3 min was selected since the difference in signal was not considered significant (ca. 2%), while the extraction time was reduced by half. This effect may be due to the rapid dispersion of the material in the solution and the ease of achieving the equilibrium. Regarding the ionic strength, the best signals were obtained at 5% (w/v) NaCl due to the salting-out effect that promotes the extraction of nonelectrolyte compounds in water. However, later, we had to readjust the salt content to 3.5% w/v NaCl because a higher salt content induced the separation of the acetonitrile from the resulting mixture obtained in the protein precipitation step when real samples were treated.

### Analytical Features of the Proposed Method

The quality parameters of the proposed method, such as linearity, limits of detection (LOD) and quantification (LOQ), and precision, both intra- and interday (expressed as relative standard deviation, RSD), were evaluated under the optimized conditions presented above.

The linearity, evaluated by measuring the working standard solutions properly treated containing a 10 ng mL^–1^ surrogate (three replicates by level), reached at least up to 450 ng mL^–1^ with coefficients of determination (*R*^2^) > 0.994.

The LOD and LOQ of the proposed method were calculated according to Eurachem Guide^40^ as 3 and 10 times, respectively, the standard deviation of a 7.5 ng mL^–1^ working standard solution prepared in synthetic saliva and subjected to the entire mSBSDME-NESI-MS/MS procedure (*n* = 10). In this sense, they were 2.25 and 7.5 ng mL^–1^, respectively.

The precision of the proposed mSBSDME method was calculated from the analysis of the working standard solutions at three different concentration levels (i.e., 7.5, 225, and 450 ng mL^–1^) properly treated and analyzed five times in the same day (intraday precision) and analyzed once in five consecutive days (interday precision). The RSD values ranged from 9 to 15%, showing satisfactory precision of the proposed method.

### Application to the Analysis of Human Saliva Samples

In order to evaluate the matrix effects by means of the relative recovery (%RR) values (eq 1), saliva samples from three nondrug consumer volunteers (two male and one female, volunteers A–C) collected as described previously were spiked at three concentration levels (i.e., 7.5, 225, and 450 ng mL^–1^) of the target analyte, and the mSBSDME-NESI-MS/MS method was applied. These results are shown in Table 1, where it can be seen that RR values were between 87 and 127%, thus showing that matrix effects were not significant and external calibration was suitable for the quantification of THC in saliva samples by the proposed method.
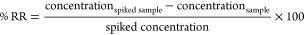
1

**Table 1:** Relative Recoveries Obtained from Samples Spiked in Different Amounts

**sample**^a^	**spiked amount(ng mL**^**–1**^**)**	**relative recovery (%)**^b^
A	7.5	100 ± 20
	225	87 ± 1
	450	100 ± 16
B	7.5	127 ± 13
	225	90 ± 11
	450	90 ± 16
C	7.5	109 ± 12
	225	93 ± 3
	450	93 ± 5

a Samples A and B: male; C: female.

b Mean of three replicates ± standard deviation.

Then, the analytical utility of the proposed method was evaluated by its application in the determination of THC in saliva samples from six marijuana smokers (four males and two females, volunteers D and I) collected as described before and analyzed by the proposed method. As can be seen in Table 2, THC was detected in all the samples, but the concentration may be variable due to the amount of marijuana present in the cigarette and smoking frequency. In any case, all the samples were positive; thus, the method would show that all these people are under the effects of a drug (i.e., THC) in the case that forensic evidence is required.

**Table 2:** Concentration Obtained by Applying the mSBSDME-NESI-MS/MS Method to Saliva Samples from Different Smokers

**volunteer**^a^	**time after smoking (h)**	**concentration(ng mL**^**–1**^**)**^b^
D	3	299 ± 5
E	1	142 ± 5
F	2	15 ± 1
G	1	445 ± 9
H	0	101 ± 9
I	0	58 ± 9

a Samples D–G: males; H and I: female.

b Mean of three replicates ± standard deviation.

### Comparison with Other Reported Methods

Compared with other reported solid- and liquid-phase extraction-based methods for the determination of THC in human saliva, the proposed mSBSDME-NESI-MS/MS method presents several advantages. As it is presented in Table 3, this approach generates a low waste volume due to the combination of the microextraction and the NESI emitter, nearly compared to the waste volume generated in the direct immersion solid-phase microextraction (DI-SPME) + thermal desorption, both much lower than the volume used in a conventional extraction technique (i.e., solid-phase extraction (SPE)). Likewise, the mSBSDME-NESI-MS/MS requires a low sample volume, which is an advantage for the analysis of low-availability samples such as saliva. In addition, the high throughput presented by the extraction assembly (up to 15 samples simultaneously) improves those values obtained by DI-SPME and microextraction by a packed sorbent (MEPS). Furthermore, the use of a magnetic sorbent provides rapid and easy handling and collection of the extraction phase.

**Table 3:** Comparison of mSBSDME with Other Extraction Approaches for the Determination of THC in Saliva

**analytes**^a^	**extraction technique**^b^	**analytical technique**^c^	**waste volume**^d^**(mL)**	**sample volume (μL)**	**sample throughput**^d^**(samples h**^**–1**^**)**	**online desorption**	**LOD**^e^**(ng mL**^**–1**^**)**	**ref.**
THC–COOH, THC–OH, **THC**, CBN, CBD	MEPS	LC-MS/MS	3	125	5	no	0.08	(41)
**THC**, CBD, CBN, and synthetic cannabinoids	DI-SPME + TD	GC-MS	1	1000	1	yes	1	(42)
**THC**, CBD	LLE	UHPLC-MS	3.75	250	11	no	0.5	(43)
THC–COOH, **THC**	automatic SPE	GC-MS	15	1000	3	no	1	(44)
**THC**	mSBSDME	NESI-MS/MS	1.7	250	10	yes	2.25	this work

a CBD: cannabidiol, CBN: cannabinol, THC: Δ^9^-tetrahydrocannabinol, THC–COOH: 11-nor-9-carboxy-Δ^9^-tetrahydrocannabinol, and THC–OH: 11-hydroxy-Δ^9^-tetrahydrocannabinol.

b DI: direct immersion, LLE: liquid–liquid extraction, MEPS: microextraction by a packed sorbent, mSBSDME: miniaturized stir bar sorptive dispersive microextraction, SPE: solid-phase extraction, SPME: solid-phase microextraction, and TD: thermal desorption.

c GC: gas chromatography, NESI: needle-based electrospray ionization emitter, LC: liquid chromatography; MS/MS: tandem mass spectrometry, and UHPLC: ultrahigh-performance liquid chromatography.

d Sample preparation + analysis.

e LOD for THC.

On the other hand, the sensitivity of the proposed method is slightly lower. This effect can be ascribed to the elution step. In conventional mSBSDME, the elution is done by dispersing the sorbent into the eluent, which guarantees a fast mass transfer. However, in this new approach, the elution is performed online while the sorbent remains attached to the stir bar. To maintain a higher sample throughput fitting the analytical purpose, only the 3 initial min of the elution profile was considered to obtain the analytical signal. Despite this effect, NESI-MS/MS facilitates the analytical procedure, avoiding additional time-consuming steps such as offline desorption. In any case, the mSBSDME-NESI-MS/MS approach provides enough sensitivity to determine the THC contents in saliva; therefore, it fits its analytical purpose.

## Conclusions

This work reports for the first time the direct coupling of mSBSDME to ambient MS. mSBSDME simplifies the typical magnetic DSPE workflow by integrating into a stirring magnet the dispersion and retrieval of the sorbent. Its miniaturized character makes mSBSDME portable and adapted to the analysis of low-availability samples. Also, the technique allows for the simultaneous extraction of several samples, thus improving the sample throughput. After the extraction, the magnet containing the sorbent and the isolated analytes is simply introduced into a stainless-steel needle, where online elution of the analytes takes place. Aided by the application of 5 kV to the needle, the eluent is electrosprayed, transferring the analytes to the MS. In this article, the bioanalytical potential of mSBSDME-NESI-MS/MS has been preliminarily evaluated by the rapid and reliable determination of THC in saliva.

The application scope of the technique is broad since it can be extended to other analytes by simply changing the magnetic sorbent. The great variety of these materials, which may include different interaction chemistries (dispersion, H-bonding, ion exchange, bioaffinity, etc.) with the analytes, supports the great versatility of the coupling. Also, the potential coupling of substrate spray techniques to portable mass spectrometers^45^ is an exciting field for further developments of mSBSDME-NESI-MS/MS.

In this preliminary approach, a slow elution of the analytes has been observed. This effect has been ascribed to the geometry of the needle hub and the positioning strategy of the stir magnet on it. Further studies will be focused on improving this elution, which should result in better sensitivity and quicker analysis.

## Supporting Information Available

Chemical structure and relevant data of the analyte (Table S1); reagents, apparatus, and experimental procedure for the synthesis of the CoFe_2_O_4_@p(DVB-co-NVP) magnetic sorbent; reagents, apparatus, and experimental procedure for preparation of synthetic saliva; reagents, apparatus, and experimental procedure for optimizing the mSBSDME variables by LC-MS/MS; liquid chromatography-tandem mass spectrometry analysis (Table S2); Plackett–Burman design (Table S3 and Figure S1); Doehlert design (Table S3) (PDF)
