# Supplementary material for: Coupling Miniaturized Stir Bar Sorptive Dispersive Microextraction to Needle-Based Electrospray Ionization Emitters for Mass Spectrometry: Determination of Tetrahydrocannabinol in Human Saliva as a Proof of Concept
Source: Anal Chem. 2024 May 14;96(23):9629–35. doi: 10.1021/acs.analchem.4c01297 (PMC11170552; doi:10.1021/acs.analchem.4c01297)
Supplement: Supplementary file 1 — ac4c01297_si_001.pdf [file ac4c01297_si_001.pdf]

SUPPORTING INFORMATION

Coupling miniaturized stir bar sorptive dispersive microextraction to needle-based electrospray ionization emitters for mass spectrometry: determination of tetrahydrocannabinol in human saliva as a proof-of-concept

Andreu L. López-Juan [a,b], Jaime Millán-Santiago [b], Juan L. Benedé [a], Alberto Chisvert [a],* Rafael Lucena [b],* Soledad Cárdenas [b]

[a] GICAPC Research Group, Department of Analytical Chemistry, University of Valencia, E-46100 Burjassot, Valencia, Spain.

[b] Affordable and Sustainable Sample Preparation (AS_2P) Research Group, Analytical Chemistry Department, Instituto Químico para la Energía y el Medioambiente (IQUEMA), University of Córdoba, Campus of Rabanales, Marie Curie Building, E-14071 Córdoba, Spain.

Corresponding authors mails: alberto.chisvert@uv.es (Prof. Dr. Alberto Chisvert), rafael.lucena@uco.es (Prof. Dr. Rafael Lucena)

Table of contents

Chemical structure and relevant data of the analyte S-1

Reagents, apparatus and experimental procedure for the synthesis of $CoFe_2O_4$@p(DVB-co-NVP) magnetic sorbent S-1

Reagents, apparatus and experimental procedure for preparation of synthetic saliva S-2

Reagents, apparatus and experimental procedure for optimizing the mSBSDME variables by LC-MS/MS S-2

Liquid chromatography-tandem mass spectrometry analysis S-4

Plackett-Burman design S-4

Doehlert design S-5

References S-6

Chemical structure and relevant data of the analyte

Table S1. Chemical structure and some relevant data of the target compound

Compound	Chemical Structure	logP_{ow}[a, b]	pKa[a, c]
(-)-trans-Δ^9-Tetrahydrocannabinol (THC)	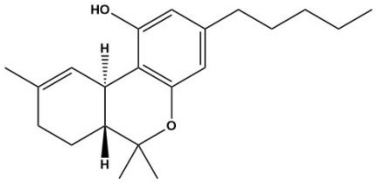	6.838	9.81

[a] Calculated using Advanced Chemistry Development (ACD/Labs) Software V11.02 (© 1994-2024 ACD/Labs)
[b] P_{ow}: Octanol-water partition coefficient.
[c] Ka: Acid dissociation constant.

Reagents, apparatus and experimental procedure for the synthesis of $CoFe_2O_4$@p(DVB-co-NVP) magnetic sorbent

Cobalt(II) chloride hexahydrate ($CoCl_2{\cdot}6H_2O$) and iron(III) chloride hexahydrate ($FeCl_3{\cdot}6H_2O$) were purchased from Acros Organics (Geel, Belgium), sodium hydroxide (NaOH) reagent grade was purchased from Scharlau (Barcelona, Spain), and divinylbenzene (DVB) 80%, N-vinyl-2-pyrrolidone (NVP) 99%, and 2,2′-azobis(2-methylpropionitrile) (AIBN) 98% were all obtained from Sigma-Aldrich (St. Louis, MO, USA). Additionally, ultrapure water (resistivity ≥ 18.2 MΩ·cm) obtained through a Connect purification system from Adrona (Riga, Latvia), ethanol LC-grade and acetonitrile LC-grade from PanReac AppliChem (Barcelona, Spain) were used as solvents.

A heating plate with a magnetic stirrer from Stuart Scientific (Staffordshire, United Kingdom) and an oven from J.P. Selecta (Barcelona, Spain) were used.

For the synthesis of the $CoFe_2O_4$ MNPs, a modified solvothermal method was carried out.[1] First, 10.80 g of $FeCl_3{\cdot}6H_2O$ (40 mmol) and 4.76 g of $CoCl_2{\cdot}6H_2O$ (20 mmol) were dissolved in 200 mL of ultrapure water, and then 100 mL of a 3 M NaOH aqueous solution were added dropwise under continuous stirring at 80 ºC for 1 h and then allowed to cool down to room temperature. The solid obtained was washed several times with ultrapure water and later with ethanol, dried at 80 ºC overnight, and ground until obtaining a fine black powder.

Once the $CoFe_2O_4$ MNPs were synthesized, they were entrapped into the copolymer chains.[2] For this purpose, 0.5 g of $CoFe_2O_4$ MNPs (2.1 mmol) and 0.1 g of AIBN (0.6 mmol) were weighed and placed in a round bottom flask. Then, 250 mL of acetonitrile, 4.45 mL of DVB (25 mmol), and 2.7 mL of NVP (25 mmol) were sequentially added, and the mixture was dispersed in an ultrasound bath for 90 min. Then, it was sealed and stirred for 24 h at 60 ºC. Afterward, the solid formed was washed three times with ethanol, and dried overnight at 80 ºC, obtaining a fine gray powder.

Reagents, apparatus and experimental procedure for preparation of synthetic saliva

NaCl 99.5% analytical grade from Fisher Scientific, potassium chloride (KCl), calcium chloride monohydrate ($CaCl_2 \cdot H_2O$), sodium dihydrogen phosphate monohydrate ($NaH_2PO_4 \cdot H_2O$) reagent grade from Scharlau, potassium thiocyanate (KSCN), urea from PanReac AppliChem, and ultrapure water, were used.

Synthetic saliva was prepared according to an adapted protocol.[3] For that aim, 250 mL of an aqueous solution containing NaCl (400 mg L^{-1}), KCl (400 mg L^{-1}), $CaCl_2 \cdot H_2O$ (795 mg L^{-1}), $NaH_2PO_4 \cdot H_2O$ (690 mg L^{-1}), KSCN (300 mg L^{-1}) and urea (1000 mg L^{-1}) in ultrapure water were prepared.

Reagents, apparatus and experimental procedure for optimizing the mSBSDME variables by LC-MS/MS

Sodium chloride (NaCl) 99.5% analytical grade used as ionic strength regulator was purchased from Fisher Scientific (Waltham, MA, USA). For the preparation of phosphate buffers, ortho-phosphoric acid (H_3PO_4) 85% reagent grade and sodium dihydrogen phosphate monohydrate ($NaH_2PO_4 \cdot H_2O$) reagent grade from Scharlau, and sodium phosphate dodecahydrate ($Na_3PO_4 \cdot 12H_2O$) from Sigma-Aldrich, were employed.

Water LC-MS grade from PanReac AppliChem, methanol (MeOH) LC-MS grade from Honeywell (Seelze, Germany) and ammonium fluoride (NH_4F) 98% from Acros Organics were used for the preparation of the chromatographic mobile phase for LC-MS/MS.

Nitrogen 99.9%, used as nebulizing and curtain gas in the MS/MS ion source, was obtained through a NiGen LCMS 40-1 nitrogen generator from Claind (Lenno, Italy). Extrapure nitrogen > 99.999% provided by Praxair (Madrid, Spain) was used as collision gas for LC-MS/MS.

An Agilent 1100 liquid chromatography (LC) system comprising a degasser, a binary pump, an autosampler, and a thermostatic column oven all from Agilent Technologies (Palo Alto, CA, USA) coupled to an Agilent 6410B Triple Quad MS/MS, was used.

The chromatographic separation was performed in a Zorbax SB-C18 (50 mm length, 2.1 mm I.D., 1.8 µm particle size) column. The injection volume was set at 5 µL, the flow rate was 0.25 mL min^{-1}, and the column temperature was kept constant at 35 °C. The mobile phase consisted of solvent A (H_2O, 0.5 mM NH_4F) and solvent B (MeOH), by isocratic elution at mixing ratio of 15:85% (v/v). The run time was 4 min.

The triple quadrupole MS detector operated in positive electrospray mode (ESI^+), at 6 kV (capillary voltage), by multiple reaction monitoring (MRM). The flow rate and temperature of the drying gas, and the nebulizer pressure were 12 L min^{-1}, 310 °C, and 35 psi, respectively. The m/z precursor → product ion transitions for identification and quantification, the collision energies, and fragmentor values for each analyte are shown in Table S2.

Liquid chromatography-tandem mass spectrometry analysis

Table S2. Tandem mass spectrometry parameters for the target analytes.

Compound	Retention time (min)	Precursor Ion (m/z)	Product ion (m/z)[a]	Collision energy (V)	Fragmentor (V)
THC	2.6	315.2	**193.1**	22	150
			123.1	34	150
			93.1	30	150
$THC-d_3$	2.6	318.2	**196.2**	26	140
			123.1	38	140
			93.1	30	140

[a] The m/z values used as quantifiers are marked in bold.

Plackett-Burman design

Table S3. Plackett-Burman design for the screening of the extraction and desorption variables.

Step	Sorbent amount, X_1 (mg)		Extraction time, X_2 (min)		pH of the donor phase, X_3		Ionic strength, X_4 (% w/v NaCl)	
	Uncoded	Coded	Uncoded	Coded	Uncoded	Coded	Uncoded	Coded
1	2.5	+1	1	-1	10	+1	0	-1
2	2.5	+1	10	+1	2	-1	10	+1
3	0.5	-1	10	+1	10	+1	0	-1
4	2.5	+1	1	-1	10	+1	10	+1
5	2.5	+1	10	+1	2	-1	10	+1
6	2.5	+1	10	+1	10	+1	0	-1
7	0.5	-1	10	+1	10	+1	10	+1
8	0.5	-1	1	-1	10	+1	10	+1
9	0.5	-1	1	-1	2	-1	10	+1
10	2.5	+1	1	-1	2	-1	0	-1
11	0.5	-1	10	+1	2	-1	0	-1
12	0.5	-1	1	-1	2	-1	0	-1

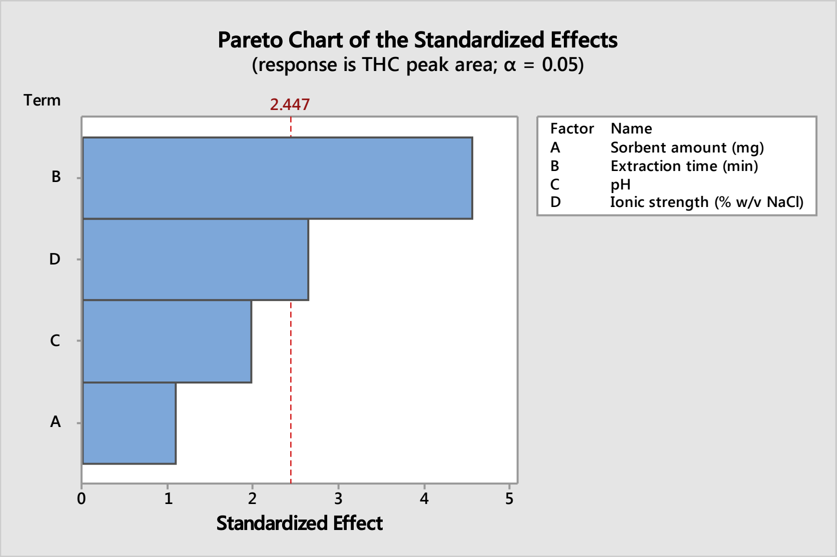

Fig. S1. Pareto chart obtained in the Plackett-Burman design.

Doehlert design

In a Doehlert design involving two variables, there are one central point and six external points forming a regular hexagon. The number of levels can differ between the variables. For example in a two-variable Doehlert design, one variable is studied at five levels while the other is studied at only three levels. This property allows a free choice of the factors to be assigned to a large or a small number of levels. Generally, it is preferable to choose the variable with the stronger effect as the factor with five levels in order to obtain most information of the system. The number of experiments required for the development of the designs (N) is defined as follows:

$$N = k^2 + k + CP \quad (1)$$

where k is the number of variables (*i.e.*, 2); and CP is the replicates of the central point (*i.e.*, 3). In this sense, according to Equation (1), 9 experiments were required, selecting 3 replicates of the central point. Table S4 showed the matrix of the experimental design of the two factors expressed as coded and uncoded values.

Table S4. Doehlert design for multivariate optimization of the critical variables.

Step	Extraction time (min)		Ionic strength (% w/v NaCl)	
	Uncoded	Coded	Uncoded	Coded
1	3.25	-0.5	0	-1
2	1	-1	5	0
3	10	1	5	0
4	7.75	0.5	10	1
5	3.25	-0.5	10	1
6	7.75	0.5	0	-1
7	5.5	0	5	0
8	5.5	0	5	0
9	5.5	0	5	0

References

(1) Maaz, K.; Mumtaz, A.; Hasanain, S. K.; Ceylan, A. Synthesis and Magnetic Properties of Cobalt Ferrite (CoFe2O4) Nanoparticles Prepared by Wet Chemical Route. *J. Magn. Magn. Mater.* **2007**, *308* (2), 289–295.

(2) Vállez-Gomis, V.; Carchano-Olcina, S.; Benedé, J. L.; Chisvert, A.; Salvador, A. Entrapment of Magnetic Nanoparticles into Poly(Divinylbenzene-Co-N-Vinylpyrrolidone) Copolymer for the Determination of Prohibited and Restricted Fragrance Ingredients in Cosmetic Products. *Microchem. J.* **2022**, *183*, 108044.

(3) Fusayama, T.; Katayori, T.; Nomoto, S. Corrosion of Gold and Amalgam Placed in Contact with Each Other. *J. Dent. Res.* **1963**, *42* (5), 1183–1197.
